# Impact of pregnancy on observed sex disparities among adults hospitalized with laboratory‐confirmed influenza, FluSurv‐NET, 2010‐2012

**DOI:** 10.1111/irv.12465

**Published:** 2017-08-01

**Authors:** Kelly Kline, James L. Hadler, Kimberly Yousey‐Hindes, Linda Niccolai, Pam D. Kirley, Lisa Miller, Evan J. Anderson, Maya L. Monroe, Susan R. Bohm, Ruth Lynfield, Marisa Bargsten, Shelley M. Zansky, Krista Lung, Ann R. Thomas, Diane Brady, William Schaffner, Gregg Reed, Shikha Garg

**Affiliations:** ^1^ Connecticut Emerging Infections Program Yale School of Public Health New Haven CT USA; ^2^ California Emerging Infections Program Oakland CA USA; ^3^ Colorado Department of Public Health and Environment Denver CO USA; ^4^ Emory University School of Medicine Atlanta GA USA; ^5^ Atlanta Veterans Affairs Medical Center Atlanta GA USA; ^6^ Maryland Department of Health and Mental Hygiene Baltimore MD USA; ^7^ Michigan Department of Health and Human Services Lansing MI USA; ^8^ Minnesota Department of Health Saint Paul MN USA; ^9^ New Mexico Department of Health Santa Fe NM USA; ^10^ New York State Department of Health Albany NY USA; ^11^ Ohio Department of Health Columbus OH USA; ^12^ Oregon Public Health Division Portland OR USA; ^13^ Rhode Island Department of Health Providence RI USA; ^14^ Vanderbilt University School of Medicine Nashville TN USA; ^15^ Utah Department of Health Salt Lake City UT USA; ^16^ Influenza Division National Center for Immunization and Respiratory Diseases CDC Atlanta GA USA

**Keywords:** hospitalization, influenza, pregnancy, relative risk, vaccination

## Abstract

**Introduction:**

Previous FluSurv‐NET studies found that adult females had a higher incidence of influenza‐associated hospitalizations than males. To identify groups of women at higher risk than men, we analyzed data from 14 FluSurv‐NET sites that conducted population‐based surveillance for laboratory‐confirmed influenza‐associated hospitalizations among residents of 78 US counties.

**Methods:**

We analyzed 6292 laboratory‐confirmed, geocodable (96%) adult cases collected by FluSurv‐NET during the 2010‐12 influenza seasons. We used 2010 US Census and 2008‐2012 American Community Survey data to calculate overall age‐adjusted and age group‐specific female:male incidence rate ratios (IRR) by race/ethnicity and census tract‐level poverty. We used national 2010 pregnancy rates to estimate denominators for pregnant women aged 18‐49. We calculated male:female IRRs excluding them and IRRs for pregnant:non‐pregnant women.

**Results:**

Overall, 55% of laboratory‐confirmed influenza cases were female. Female:male IRRs were highest for females aged 18‐49 of high neighborhood poverty (IRR 1.50, 95% CI 1.30‐1.74) and of Hispanic ethnicity (IRR 1.70, 95% CI 1.34‐2.17). These differences disappeared after excluding pregnant women. Overall, 26% of 1083 hospitalized females aged 18‐49 were pregnant. Pregnant adult females were more likely to have influenza‐associated hospitalizations than their non‐pregnant counterparts (relative risk [RR] 5.86, 95% CI 5.12‐6.71), but vaccination levels were similar (25.5% vs 27.8%).

**Conclusions:**

Overall rates of influenza‐associated hospitalization were not significantly different for men and women after excluding pregnant women. Among women aged 18‐49, pregnancy increased the risk of influenza‐associated hospitalization sixfold but did not increase the likelihood of vaccination. Improving vaccination rates in pregnant women should be an influenza vaccination priority.

## 1 INTRODUCTION

A monograph on sex and gender differences in influenza published by the World Health Organization in 2010 1 concluded that the “rates of hospitalization from seasonal influenza viruses are consistently larger in males than female of all ages, where data are available.” This conclusion was based upon studies of hospital discharge data in Canada 2 and Denmark.^3^ In contrast, these studies showed that women of reproductive age had rates similar to or slightly higher than males of the same age. These studies were limited, however, in that they did not specifically examine hospitalization rates among confirmed influenza cases; rather, they examined hospitalization discharge codes for pneumonia and influenza.

In 2003, the US Centers for Disease Control and Prevention established population‐based enhanced surveillance for laboratory‐confirmed influenza‐associated hospitalization, FluSurv‐NET.^4^ First initiated in 10 states as part of the Emerging Infections Program (EIP), FluSurv‐NET expanded during the 2009 influenza pandemic to include an additional four sites. The first FluSurv‐NET publication on influenza hospitalizations in adults, covering 2005‐2008, found that 57% of adult cases were female and that, even after excluding pregnant females, more than half (55%) were female.^5^ A more detailed analysis of differences in influenza‐associated hospitalization incidence by sex over a seven‐year time period was performed with data from a single FluSurv‐NET site, Connecticut.^6^ Female sex was independently associated with higher rates of hospitalization, even after controlling for pregnancy. This finding was most prominent among women of high census tract‐level poverty, blacks, and Hispanics. It could not be explained by pregnancy, vaccination rates, or underlying comorbidities.

No studies have comprehensively examined these factors across a large geographic area in the United States during non‐pandemic influenza seasons to determine whether these findings could be applied more broadly. To better characterize observed sex differences, we used data from FluSurv‐NET sites that conducted enhanced population‐based surveillance during the 2010‐2011 and 2011‐2012 influenza seasons for laboratory‐confirmed influenza‐associated hospitalizations.

## 2 METHODS

We used laboratory‐confirmed adult (≥18 years) influenza‐associated hospitalizations reported through enhanced population‐based surveillance by the 14 FluSurv‐NET sites during the 2010‐11 and 2011‐12 influenza seasons. These cases had been previously geocoded for an analysis of influenza census tract poverty levels.^7^ The sites, located in California, Colorado, Connecticut, Georgia, Maryland, Michigan, Minnesota, New Mexico, New York, Ohio, Oregon, Rhode Island, Tennessee, and Utah, included 78 counties with a total catchment population of nearly 21 million adults. FluSurv‐NET monitors laboratory‐confirmed influenza‐associated hospitalizations defined as hospitalization of persons residing in the surveillance area ≤14 days after or ≤ 3 days before a positive influenza test result during October 1‐April 31 each year. Cases are identified by actively determining whether persons with positive influenza tests reported by clinical laboratories were hospitalized. Individual data are collected through review of medical records. Vaccination status is collected through review of medical records, vaccine registries, primary care provider, or interview of patients or their proxies. Descriptions of FluSurv‐NET sites and data collection have been described elsewhere.^4^, ^8^

### 2.1 Census data

Overall and group‐specific population estimates for the residential catchment areas were obtained from the 2010 US Census and used for the denominator in all incidence calculations. Neighborhood SES data were obtained from the 2008‐2012 American Community Survey and used four categories describing the percentage of households living below the federal poverty level in each census tract (<5%, 5‐<10%, 10‐<20%, and ≥20%), as recommended by the Public Health Disparities Geocoding Project 9, 10 and used in previous FluSurv‐NET analyses of influenza data.^6^, ^7^, ^11^, ^12^

### 2.2 Data analysis

Statistical analyses were performed using SAS version 9.3 (SAS Institute Inc, Cary, NC, USA). Female and male average annual incidence rates per 100 000 persons were calculated by dividing the total number of cases over the two seasons by twice the total number of adults in the residential catchment area. Female:male incidence rate ratios (IRR) were calculated by dividing the incidence rate among females by the incidence rate among males. IRRs were also calculated by age group (18‐49, 50‐64, 65‐74, 75‐84, and ≥85 years old), race/ethnicity (non‐Hispanic white, non‐Hispanic black, Hispanic, non‐Hispanic Asian, and non‐Hispanic other races), SES group, and FluSurv‐NET site. All incidence rates except the age group‐specific ones were age‐adjusted using the 2000 US Standard Population and the age groups specified above.

Incidence rates were also calculated for women 18‐49 years old excluding pregnant cases, and new female:male IRRs were then determined. Pregnant cases were excluded from the numerator, and the estimated time pregnant women contributed was excluded from the denominator. As some women could be both pregnant and non‐pregnant during the same 7‐month surveillance period, we used person‐weeks during the 7 months (30 weeks) of surveillance for the denominators for this comparison. The total number of person‐weeks contributed by all women was the total number of women in this age group times 30. We assumed the pregnancy rates of all women 18‐49 years overall, by age group, and by race/ethnicity who were pregnant in 2010 were the same in the catchment area as in the USA as a whole.^13^ Of note, these annual pregnancy rates are only an approximation of the number of pregnant women in the population, as a woman may experience multiple pregnancy outcomes over the surveillance period and there may be women who experience a pregnancy with an unreported abortion or miscarriage. As a woman would only be pregnant for 40 of the 52 weeks in a year, we multiplied the estimated number of pregnant women by 40/52 to adjust for this and then multiplied by 30 to determine their contribution in person‐weeks to the total. We further adjusted this to account for shorter pregnancies due to abortions and miscarriages. Based on the 2009 national pregnancy report,^14^ 65% of pregnancies resulted in live births and were given credit for term pregnancies. The 35% that resulted in abortion or miscarriage were given credit for an average of 20 weeks of pregnancy.

Age group and race/ethnicity differences in percentage of pregnancies resulting in live births 14 were accounted for in age group‐specific and race/ethnic group‐specific analyses. The total number of person‐weeks contributed by all men for the comparison was the total number of men in this age group multiplied by 30.

We also compared the influenza hospitalization rate for 18‐ to 49‐year‐old pregnant women to the rate in non‐pregnant women using IRRs calculated based on person‐weeks. The numerators were the pregnant and non‐pregnant cases diagnosed during each surveillance season, and the denominators were those for each season as calculated above.

## 3 RESULTS

Overall, 96% of the cases were able to be geocoded, resulting in a dataset with 6292 hospitalized adults with laboratory‐confirmed influenza. Of these, 55.5% were female, more than 30% were aged 18‐49, and the majority were white (61.5%) (Table 1). Additionally, higher percentages of cases lived in poorer census tracts.

**Table 1:** Characteristics of adult cases with laboratory‐confirmed influenza‐associated hospitalizations, FluSurv‐NET, 2010‐2012

Characteristic	Number (n=6292)^a^	Percent^b^
Sex		
Female	3493	55.5
Male	2799	44.5
Age		
18‐49	1897	30.1
50‐64	1498	23.8
65‐74	899	14.3
75‐84	1026	16.3
≥85	972	15.4
Race/Ethnicity		
White	3554	61.5
Black	1254	21.7
Hispanic	597	10.3
Asian	298	5.2
Other	76	1.3
Poverty Level by Census Tract		
<5%	1239	19.7
5‐<10%	1434	22.8
10‐<20%	1735	27.6
≥20%	1880	29.9

a Numbers may not add due to missing data; missing 513 (8.2%) race/ethnicity and four (<1%) poverty level by census tract.

b Numbers may not total 100% due to rounding.

Adult females were overall 1.17 (95% CI, 1.11 ‐ 1.22) times more likely to be hospitalized than males (Table 2). The higher overall IRR among females was almost exclusively driven by higher incidence rates among females than males aged 18‐49 (IRR 1.32, 95% CI 1.20 ‐ 1.44). The incidence rate ratio was not different for females and males in each subsequent age group with the exception of females having an IRR statistically significantly lower than that of males beginning at age 75.

**Table 2:** Number of cases, incidence rates, and female:male incidence rate ratios (IRRs) of laboratory‐confirmed influenza‐associated hospitalizations by age and sex, FluSurv‐NET, 2010‐2012

	Cases	Incidence per 100 000 person‐years	IRR	95% CI	*P*
Overall					
Female	3493	32.32	1.17	1.11, 1.22	<0.001
Male	2799	27.73	Ref	—	—
18‐49					
Female	1083	17.07	1.32	1.20, 1.44	<0.001
Male	814	12.97	Ref	—	—
50‐64					
Female	793	29.75	1.05	0.95, 1.65	0.33
Male	705	28.27	Ref	—	—
65‐74					
Female	473	52.14	0.95	0.83, 1.08	0.42
Male	426	55.05	Ref	—	—
75‐84					
Female	570	97.69	0.87	0.77, 0.99	0.03
Male	456	111.86	Ref	—	—
≥85					
Female	574	187.90	0.67	0.59, 0.77	<0.001
Male	398	279.05	Ref	—	—

In unadjusted comparisons among 18‐ to 49‐year‐olds, females of all three major race/ethnicity groups and those living in census tracts with ≥10% poverty were significantly more likely to be hospitalized than males in the same race/ethnicity group or within the same poverty level (Table 3). In particular, Hispanic females aged 18‐49 were 70% more likely to be hospitalized than Hispanic males in this age group. Females aged 18‐49 living in census tracts with ≥10% poverty levels were at least 1.4 times more likely than their male counterparts to be hospitalized. When pregnant women were removed from the analysis, there were no statistical differences in IRRs by sex for 18‐ to 49‐year‐olds for all subgroups defined by race/ethnicity or by census tract poverty.

**Table 3:** Female:male incidence rate ratios (IRR) of laboratory‐confirmed influenza‐associated hospitalizations among 18‐ to 49‐year‐olds with and without pregnant females by race/ethnicity and census tract poverty level, FluSurv‐NET, 2010‐2012

	Cases	IRR	95% CI	*P*	IRR excluding pregnant females	95% CI	*P*
Total (aged 18‐49)							
Female	1083	1.17	1.11, 1.22	<0.001	1.03	0.94, 1.14	0.51
Male	814	Ref	—	—			
Race/Ethnicity							
White, non‐Hispanic							
Female	423	1.16	1.01, 1.34	0.03	0.96	0.83, 1.12	0.64
Male	366	Ref	—	—	Ref	—	—
Black, non‐Hispanic							
Female	338	1.26	1.07, 1.49	0.006	1.01	0.85, 1.21	0.88
Male	236	Ref	—	—	Ref	—	—
Hispanics							
Female	169	1.70	1.34, 2.17	<0.001	1.11	0.85, 1.45	0.46
Male	109	Ref	—	—	Ref	—	—
Census Tract Poverty Level							
<5% Poverty Census Tracts							
Female	137	1.10	0.86, 1.41	0.43	0.87	0.67, 1.14	0.31
Male	120	Ref	—	—	Ref	—	—
5‐<10% Poverty Census Tracts							
Female	182	1.05	0.85, 1.29	0.67	0.87	0.70, 1.09	0.23
Male	169	Ref	—	—	Ref	—	—
10‐<20% Poverty Census Tracts							
Female	319	1.42	1.19, 1.68	<0.001	1.10	0.91, 1.32	0.32
Male	222	Ref	—	—	Ref	—	—
≥20% Poverty Census Tracts							
Female	444	1.50	1.30, 1.74	<0.001	1.15	0.99. 1.35	0.07
Male	301	Ref	—	—	Ref	—	—

Overall, 282 (26%) of 1083 hospitalized females aged 18‐49 were reported to be pregnant at the time of hospitalization. Pregnant women were nearly six times more likely to have an influenza‐associated hospitalization than non‐pregnant women aged 18‐49. Although the relative risk of hospitalization of pregnant women decreased with increasing age, it was higher in all age groups (Table 4). Of the 282 pregnant cases, 269 (95%) had information on trimester of pregnancy at the time of hospitalization: 23 (8.6%) were in their first trimester, 84 (31.2%) were in their second, and 162 (60.2%) were in their third. These relative percentages by trimester did not differ significantly when the women were split into 5‐year age groups.

**Table 4:** Relative rates of hospitalization with influenza among 18‐ to 49‐year‐old females with laboratory‐confirmed influenza‐associated hospitalizations by age and pregnancy status, FluSurv‐NET, 2010‐2012

Age	Cases	Pregnant Cases	IRR^a^	95% CI	*P*
18‐19	42	19 (45.2%)	13.04	7.10, 23.94	<0.001
20‐24	196	93 (47.4%)	9.06	6.84, 11.99	<0.001
25‐29	208	85 (40.9%)	6.08	4.61, 8.01	<0.001
30‐34	163	51 (31.3%)	4.62	3.32, 6.43	<0.001
35‐39	151	27 (17.9%)	4.41	2.91, 6.68	<0.001
40‐49^b^	323	7 (2.2%)	3.78	1.79, 8.00	<0.001
Total	1083	282 (26.0%)	5.86	5.12, 6.71	<0.001

Ratio of incidence in pregnant women:non‐pregnant women.

Women aged 40‐44 and 45‐49 were collapsed into one group due to lack of pregnant admissions in the 45‐ to 49‐year age group.

Despite the elevated risk of influenza hospitalization, pregnant female cases aged 18‐49 were no more likely to have been vaccinated than non‐pregnant females aged 18‐49 (25.5% vs 27.8%, *P*=0.48, Table 5). The overall vaccination rate in this age group was much lower than the 51.9% found in female cases older than 50 years.

**Table 5:** Vaccine coverage among female influenza‐associated hospitalizations aged 18‐49 by pregnancy status^a^, FluSurv‐NET, 2010‐2012

Age	Pregnant		Not Pregnant		*P* b
	Cases	Number vaccinated (%)	Cases	Number vaccinated (%)	
18‐24	99	25 (25.3%)	107	26 (24.3%)	0.87
25‐29	74	11 (14.9%)	107	30 (28.0%)	0.04
30‐34	42	15 (35.7%)	100	26 (26.0%)	0.24
35‐39	25	11 (44.0%)	108	26 (24.1%)	0.05
40‐49	7	1 (14.3%)	272	85 (31.3%)	0.33
Total	247	63 (25.5%)	694	193 (27.8%)	0.48

a There were 142 (13.1%) females aged 18‐49 with missing vaccination status.

b *P*‐values were calculated by chi‐square for age groups 18‐24, 25‐29, 30‐34, and 35‐39. A Fisher's exact test was used for aged 40‐49.

While the IRR of influenza‐associated hospitalization among pregnant women was similar across the 14 FluSurv‐NET sites, the vaccination rates of pregnant cases varied widely (range: 9.1‐57.1%, *P*<0.01, data not shown).

## 4 DISCUSSION

We had several notable findings. First, we found that adult females overall were at greater risk for influenza‐associated hospitalizations than males (IRR 1.17, 95% CI 1.11 ‐ 1.22). However, this result was driven almost entirely by the 18‐ to 49‐year age group. In contrast, males aged ≥75 years were at significantly greater risk of being hospitalized with influenza compared to females of the same age. Second, within the 18‐ to 49‐year age group, Hispanic, non‐Hispanic black, and non‐Hispanic white women and women living in census tracts with ≥10% poverty levels were at significantly greater risk than their male counterparts. However, these significant differences disappeared when pregnant women were excluded from analysis. Third, pregnant women aged 18‐49 were at nearly sixfold higher risk for hospitalization than women aged 18‐49 who were not pregnant. Finally, we found that pregnant cases were no more likely than their non‐pregnant age‐matched counterparts to be vaccinated against influenza.

In contrast to the WHO monograph, we found few differences in hospitalization rates with influenza between adult males and females. Only females aged 18‐49 and older males (≥75 years) were at significantly greater risk for hospitalization with influenza. Importantly, our study was exclusively based on laboratory‐confirmed influenza cases while the data cited in the monograph used hospital discharge codes for pneumonia and influenza. It is likely that causes of pneumonia other than influenza could impact the incidence rates presented in these studies.

The finding that excluding women who were pregnant explained sex differences in this study is different than that found in the earlier multiyear site‐specific study in Connecticut using the same surveillance system.^6^ However, there are several differences between the two analyses. First, the former EIP study only analyzed data from one site. Second, the current analysis covered two years of influenza‐associated hospitalization surveillance during which H3N2 was the predominant circulating influenza A virus and accounted for most cases. In contrast, the Connecticut study covered four seasons of influenza‐associated hospitalizations, including the 2009 pandemic H1N1 strain which affected younger age groups and has been shown to have a high relative hospitalization rate for pregnant women.^15^

The sixfold elevated risk of hospitalization among pregnant women compared to non‐pregnant women aged 18‐49 is consistent with previous estimates of the additional risk of hospitalization from pandemic influenza.^15^ It is also consistent with the increased risk of hospitalization for pregnant women during the influenza season compared to other times of year.^16^, ^17^ We were unable to find other estimates of the magnitude of increased risk during seasonal influenza other than these ecologic studies. This study is the first, to our knowledge, to address the magnitude of the increased risk during pregnancy for seasonal influenza using an influenza‐specific surveillance system. The finding that most hospitalized pregnant women were in their third trimester is also consistent with previous studies.^17^, ^18^ The main reasons pregnant women are at higher risk for severe illness with influenza are thought to relate to physiologic changes during pregnancy. These changes include increases in heart rate, stroke volume, and oxygen consumption, decreases in lung capacity, and changes in immunologic function during pregnancy, all of which peak during the third trimester.^19^ Another possible contributing factor that we were unable to investigate is that clinicians may have a lower threshold for testing and admitting pregnant women near the end of their pregnancy to closely monitor the impact of maternal illness on the pregnancy and fetus.

We compared the rate of vaccination among pregnant to non‐pregnant cases as a surrogate for the relative rate of vaccination among pregnant and non‐pregnant women 18‐49 years. Assuming vaccination effectiveness was the same in each group, then the relative rate between cases should reflect the relative rate among the population groups from which the cases came. While we expected to find that pregnant cases would have higher vaccination rates than non‐pregnant case women, they did not. Although recommendations for vaccination of all women of childbearing age regardless of risk factors were only first made in 2010,^20^ vaccination recommendations targeting pregnant women have been in place since 1997.^21^ Further, the 2009‐2010 pandemic season that preceded the two seasons examined in this study clearly demonstrated that pregnant women were at high risk of complications.^15^ Thus, we found it surprising that vaccination rates in cases were very similar among both pregnant and non‐pregnant women.

The finding that pregnant women were at a much higher risk of hospitalization but not more likely to be vaccinated reinforces long‐standing vaccine policy recommendations for focused efforts targeting pregnant women regardless of their stage of pregnancy.^22^ There are several important reasons to vaccinate pregnant women: First, they are at higher risk of complications and severe illness when they get influenza. Secondly, they provide passive immunity to protect their future infant.^23^ Finally, by receiving vaccination, they may be less likely to become influenza infected and transmit it to their infants.

Recommendations in the United States specifically encouraging vaccination of pregnant women regardless of stage of pregnancy began in 2004. After 2004, vaccination rates of pregnant women rose slowly from a baseline of 10%‐21%, but remained low through at least 2007.^24^ During and following the 2009 H1N1 influenza A pandemic, rates continued to rise, reaching 46% for monovalent H1N1 vaccine and 32% for seasonal influenza vaccine in 2009‐10 25 and 44% for seasonal influenza vaccine during 2010‐11, when our study began.^26^ The 2010‐11 rate for pregnant women was higher than the 25.5% rate we observed in pregnant female cases. The difference in vaccination rates between cases and all pregnant women is expected, as vaccination should prevent some vaccinated women in each group from being admitted.

Regardless of the differences in vaccine coverage rates, national estimates still fall short of the Healthy People goal of 80% coverage.^27^ To address this, additional efforts were recently taken to make sure all clinicians who provide care to pregnant women are aware of recommendations and their role in assuring all are offered influenza vaccine.^28^ These efforts have included a public information campaign launched in 2014 by the CDC and partner organizations targeted at healthcare providers who provide clinical services to pregnant women, including sending a Dear Clinician letter to licensed healthcare providers in the USA.^29^

This study has several strengths. First, it is a population‐based study of laboratory‐confirmed influenza cases obtained through enhanced surveillance. While some other studies of influenza in females were population‐based, they did not use laboratory‐confirmed influenza cases.^2^, ^3^ Second, this study is more robust in geographic and population coverage, including data from 14 states, representing a population of nearly 21 million adults, and thus, provides a picture that is more generalizable than one from a single state or hospital. Third, we systematically collected vaccine history data, enabling us to compare influenza vaccination rates among pregnant cases to non‐pregnant case women of the same age groups. Recent reports of vaccination rates in pregnant women have not included information on non‐pregnant women.^26^, ^30^ This surveillance system has the potential to continue to provide information on the relative success of efforts to get pregnant women vaccinated against influenza compared to age‐matched non‐pregnant women.

This study also has several notable limitations. First, analyses covered two flu seasons, 2010‐2011 and 2011‐2012, which are not fully representative years of influenza hospitalization patterns. In particular, the 2011‐2012 season had a much later onset and much lower collective morbidity than usual and H3N2 was the predominant influenza A virus. Analyses from different years are needed to determine the consistency of these findings. This is especially important given that the circulating strains in a given year may affect sex differences, especially if there is little existing immunity in the population. Second, conclusions cannot be extrapolated beyond hospitalization to true influenza incidence or mortality patterns in the population, as the reasons for hospitalization and influenza testing, especially of pregnant women, may reflect other issues in addition to influenza. Such issues include a possibly lower patient threshold for seeking healthcare services, and a lower provider threshold for hospitalization for patient observance, particularly in the third trimester. Third, there were assumptions made in the methods used. National pregnancy rates were age‐ and race/ethnicity‐specific, but there were not pregnancy rates by census tracts. Although our catchment area was widely distributed throughout the USA, available national rates may not have reflected the real pregnancy rates in these 14 sites. We also potentially overestimated the pregnant‐weeks denominator by allotting 20‐week gestation to all documented miscarriages, despite most being in the first trimester. In addition, we allotted 40 weeks gestation to all live births even though many were born prematurely. The effect of this would be to underestimate the risk during pregnancy, making the overall relative risk of influenza‐associated hospitalization during pregnancy of nearly six an underestimate. It is also possible that some pregnant women were not identified as being pregnant, although this would be most likely among first‐trimester pregnancies, which contribute little to the pregnancy‐weeks denominator. In the same vein, we did not account for the possibility that a woman who delivered during the surveillance period became pregnant again later in the surveillance period. We estimate that as many as 3.4% of those who delivered became pregnant again during the same influenza surveillance season. Not counting them in the pregnant‐weeks denominator could result in a slight overestimation of the risk of influenza‐associated hospitalization. Additionally, there were no data available to account for differences in testing practices between hospitals and/or 14 FluSurv‐NET sites. Finally, some data were missing, including census tract of residence for 4% of cases and vaccination status for 13% of women 18‐49 years old.

In summary**,** this is the first study to assess sex differences in laboratory‐confirmed influenza‐associated hospitalizations across a large geographic area with many cases. This study provides new evidence that in the United States from 2010 to 2012, sex differences in influenza‐associated hospitalizations were driven by pregnancy. Additionally, it confirms a high relative risk of hospitalization of pregnant women during two interpandemic seasons where H3N2 influenza was the predominant influenza A virus (seasonal influenza) and found that hospitalized pregnant women in 2010‐2012 were no more likely to have been vaccinated than hospitalized non‐pregnant women of the same age. These results reinforce the importance of policies targeting pregnant women for influenza vaccination efforts.

## CONFLICT OF INTEREST

None of the authors has competing financial interests to disclose.
